# HBV continuum of care using community- and hospital-based screening interventions in Senegal: Results from the PROLIFICA programme

**DOI:** 10.1016/j.jhepr.2022.100533

**Published:** 2022-07-09

**Authors:** Amina Sow, Maud Lemoine, Papa Souleymane Toure, Madoky Diop, Gora Lo, Jean De Veiga, Omar Thiaw Pape, Khady Seck, Gibril Ndow, Lamin Bojang, Arame Kane, Marina Oudiane, Jess Howell, Shevanthi Nayagam, Jude Moutchia, Isabelle Chemin, Maimuna Mendy, Coumba Toure-Kane, Mark Thursz, Mourtalla Ka, Yusuke Shimakawa, Souleymane Mboup

**Affiliations:** 1 Institut de Recherche en Santé de Surveillance Epidemiologique et de Formation (IRESSEF) Laboratoire CHNU Dalal Jamm Guediawaye, IRESSEF Diamnoadio Dakar, Senegal; 2 Laboratoire de Virology, Hopital Le Dantec, Dakar, Senegal; 3 Department of Metabolism, Digestion and Reproduction, Division of Digestive Diseases, Section of Hepatology, St Mary’s Hospital, Imperial College London, London, UK; 4 Medical Research Council the Gambia Unit at the London School of Hygiene and Tropical Medicine, Fajara, The Gambia; 5 UFR des Sciences de la Sante, Thies, Senegal; 6 Centre hospitalier de Tivaoaune, Service de Medecine interne, Thies, Senegal; 7 Hopital Saint Jean de Dieu, Service d’Hepatologie et Gastroenterologie, Thies, Senegal; 8 Hopital Saint Jean de Dieu, Laboratoire d’analyse biochimique et hématologique, Thies, Senegal; 9 Centre hospitalier régional de Thies, Service de Medecine interne, Thies, Senegal; 10 Disease Elimination, Burnet Institute, Department of Gastroenterology, St. Vincent's Hospital Department of Epidemiology and Preventive Medicine, Monash University Melbourne, Melbourne, Victoria, Australia; 11 MRC Centre for Global Infectious Disease Analysis, School of Public Health, Imperial College London, London, UK; 12 Unité d'Épidémiologie des Maladies Émergentes, Institut Pasteur Paris, France; 13 INSERM U1052, CNRS 5286, Université Lyon, Université Claude Bernard Lyon 1, Centre Léon Bérard, Centre de Recherche en Cancérologie de Lyon, F-69000, Lyon, France; 14 International Agency for Research on Cancer (IARC), Lyon, France

**Keywords:** Hepatitis B, Africa, Screening, Diagnosis, Treatment, ALP, alkaline phosphatase, ALT, alanine transaminase, aOR, adjusted odds ratio, APRI, AST-to-platelet ratio index, AST, aspartate aminotransferase, cOR, crude odds ratio, eGFR, estimated glomerular filtration rate, GGT, gamma-glutamyl transferase, HBsAg, hepatitis B surface antigen, HCC, hepatocellular carcinoma, LSM, liver stiffness measurement, POC, point of care, PROLIFICA, Prevention of Liver Fibrosis and Cancer in Africa, qPCR, quantitative polymerase chain reaction, sSA, sub-Saharan Africa, TDF, tenofovir disoproxil fumarate, WHO, World Health Organization

## Abstract

**Background & Aims:**

Strategies to implement HBV screening and treatment are critical to achieve HBV elimination but have been inadequately evaluated in sub-Saharan Africa (sSA).

**Methods:**

We assessed the feasibility of screen-and-treat interventions in 3 real-world settings (community, workplace, and hospital) in Senegal. Adult participants were screened using a rapid HBsAg point-of-care test. The proportion linked to care, the proportion who had complete clinical staging (alanine transaminase [ALT], viral load, and FibroScan®), and the proportion eligible for treatment were compared among the 3 intervention groups.

**Results:**

In 2013–2016, a total of 3,665 individuals were screened for HBsAg in the community (n = 2,153) and in workplaces (n = 1,512); 199/2,153 (9.2%) and 167/1,512 (11%) were HBsAg-positive in the community and workplaces, respectively. In the hospital setting (outpatient clinics), 638 HBsAg-positive participants were enrolled in the study. All infected participants were treatment naïve. Linkage to care was similar among community-based (69.9%), workplace-based (69.5%), and hospital-based interventions (72.6%, *p* = 0.617). Of HBV-infected participants successfully linked to care, full clinical staging was obtained in 47.5% (66/139), 59.5% (69/116), and 71.1% (329/463) from the community, workplaces, and hospitals, respectively (*p* <0.001). The proportion eligible for treatment (EASL criteria) differed among community- (9.1%), workplace- (30.4%), and hospital-based settings (17.6%, *p* = 0.007). Acceptability of antiviral therapy, adherence, and safety at 1 year were very good.

**Conclusions:**

HBV screen-and-treat interventions are feasible in non-hospital and hospital settings in Senegal. However, the continuum of care is suboptimal owing to limited access to full clinical staging. Improvement in access to diagnostic services is urgently needed in sSA.

**Lay summary:**

Hepatitis B infection is highly endemic in Senegal. Screening for infection can be done outside hospitals, in communities or workplaces. However, the hepatitis B continuum of care is suboptimal in Senegal and needs to be simplified to scale-up diagnosis and treatment coverage.

## Highlights

• Strategies to improve diagnosis and treatment for hepatitis B are urgently needed in Africa.
• HBV case finding is possible outside the hospitals (communities and workplaces) in Senegal.
• Linkage to care and acceptance of antiviral therapy are good in patients screened in non-hospital and hospital-based settings.
• The HBV continuum of care is highly affected by obtaining a full clinical staging in Senegal.
• Improvement in access to diagnostic services is urgently needed in sub-Saharan Africa.

## Graphical abstract

**Figure:**
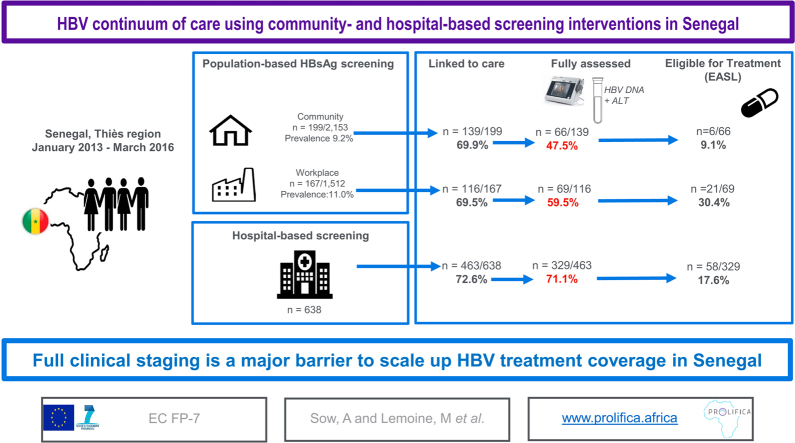

## Introduction

By 2040, the number of deaths from chronic viral hepatitis worldwide is projected to exceed those from HIV, tuberculosis, and malaria.^1^ In 2016, the United Nations General Assembly and the World Health Organization (WHO) adopted an ambitious global strategy that aimed to eliminate viral hepatitis by 2030. This elimination plan will require a substantial increase in diagnosis and antiviral treatment coverage.[2], [3], [4]

With 80 million people chronically infected with HBV and nearly 100,000 HBV-related annual deaths, sub-Saharan Africa (sSA) ranks as one of the most affected regions by HBV worldwide.^5^^,^^6^ In most African countries, HBV vaccine was only introduced in the late 2000s in the form of a combined vaccine starting at 6–8 weeks of life, without birth dose, and its coverage remains inadequate. Moreover, screen-and-treat strategies targeting adults living with chronic HBV infection, aimed at reducing the risk of HBV related mortality, have not been prioritised in the public health agenda of African countries.[7], [8], [9], [10], [11] The 2019 estimates of HBV diagnosis and treatment are extremely low in sSA, where only 2% of HBV-infected people are diagnosed and 0.1% treated when necessary.^6^^,^^12^ As a result, no African country is currently on track to eliminate HBV by 2030.^13^ Strategies to fill this gap have been poorly evaluated in sSA. A population-based study conducted by our group in The Gambia suggested that a community-based screen-and-treat intervention for HBV infection is feasible and cost-effective.^7^^,^^14^ However, whether and how such interventions can be implemented in real-world settings in sSA with limited local infrastructure remains to be evaluated on a country level.^13^

Senegal is one of the most endemic countries for HBV infection worldwide, with an estimated hepatitis B surface antigen (HBsAg) prevalence of around 10%.^15^^,^^16^ Studies on hepatitis B in Senegal are, however, mainly sero-surveys conducted in selected populations and rarely report the proportion of HBV-infected individuals in need of antiviral therapy or do not provide information on the HBV continuum of care.^17^ However, such data are critical to inform national and regional hepatitis programmes and to develop evidence-based strategies adapted to the local epidemiology and available resources.

In 2013, the Prevention of Liver Fibrosis and Cancer in Africa (PROLIFICA) programme set up the first HBV screen-and-treat intervention in Senegal. Here, we report the feasibility of screen-and-treat interventions in 3 different settings, namely, community-based, workplace-based, and hospital-based, through the assessment of the HBV continuum of care in the region of Thiès, Senegal.

## Patients and methods

### Screening sites

There were 3 screening settings: communities, workplaces, and hospitals. To align with the intervention in The Gambia^7^ for the community setting, all adults aged 30 years or older who signed a consent form were eligible for screening. In workplaces and hospitals, there was no age restriction for screening.

Community-based screening was conducted in 11 districts (7 rural and 4 urban communities) in the region of Thiès. We first organised a sensitisation meeting with community and religious leaders and obtained community approval to undertake HBV screening in their communities. At the screening session, pretest counselling was followed by HBsAg screening using a rapid point-of-care (POC) test (Determine®, Alere, Waltham, USA).^18^ Results were provided on site with post-test counselling, and those who tested positive were referred to the liver clinic of 1 of the 3 study hospitals described below.

For the workplace screening, the 5 largest chemical factories in the region were invited to participate in this study. We first explained the study to the manager of each factory and their occupational physicians. Then they informed and invited the employees to undertake screening for HBsAg, which was performed by the study team. Each employee who tested positive was then referred to the closest liver clinic out of the 3 study hospitals for further clinical assessment as described above.

Hospital-based screening was carried out in the 3 main hospitals in Thiès (Regional Hospital, Saint Jean de Dieu Hospital, and Barthimée Hospital) and targeted patients attending the outpatient digestive disease services without a known history of HIV infection. Those who tested positive for HBsAg using the Determine® POC test were referred for further clinical assessment.

### Linkage to care

Linkage to care was defined as at least 1 attendance to the outpatient liver clinic following a positive HBsAg test. HBsAg-positive participants who did not attend the clinic were systematically reminded by a study nurse via up to 2 phone calls. Transportation fees to attend clinical appointments were covered by the study.

### Liver assessment

A standardised liver assessment was performed at the study clinic: physical examination, abdominal ultrasound, fasting liver stiffness measurement (LSM) using transient elastography (FibroScan®, FS502 Echosens®, Paris, France).^19^ Only 1 FibroScan® machine was accessible at Thiès University.

We used 7.9 and 9.5 kPa as LSM cut-off values for significant fibrosis (METAVIR score ≥F2) and cirrhosis (F4), respectively.^20^ These cut-offs were determined by a previous validation study in The Gambia, where the sensitivity and specificity to predict cirrhosis were 100% and 89%, respectively.^20^

Excessive alcohol intake was estimated as more than 30 g/day for men and 20 g/day for women through a questionnaire administered to study participants. Participants were seen by a study nurse and a liver specialist. Clinical and laboratory costs were covered by the study.

### Laboratory analysis

During the first clinical visit, participants had biochemical (liver transaminases) and haematological (full blood count) tests and the following serological tests: HBeAg and antibody to HBeAg (anti-HBeAb; Architect i1000 SR, Abbott, North Chicago, IL, USA), HIV-1 and HIV-2 antibodies (EIA, Genscreen ULTRA HIV Ag-Ab, Bio-Rad, Hercules, CA, USA), and anti-HDV (ETI-AB-DELTAK-2, DiaSorin, Saluggia VC, italy) were analysed in a subgroup of participants. HBV DNA was quantified using an in-house real-time PCR (detection limit 50 IU/ml), the accuracy of which was validated against a commercial quantitative PCR (qPCR; Abbott, Wiesbaden, Germany).^21^

### Treatment eligibility

To select patients for antiviral therapy, the EASL treatment criteria were applied.^22^ In the absence of contraindications, tenofovir disoproxil fumarate (TDF; Gilead Sciences, Foster City, USA), one 300-mg pill per day, was provided free of charge. Adherence to treatment was assessed by clinicians every 3 months using the Morisky scale.^23^

### Statistical analysis

In this analysis, we had 4 continuum-of-care outcomes: (i) the proportion of people who tested positive for HBsAg; (ii) the proportion of HBsAg-positive people who were successfully linked to care; (iii) the proportion of HBsAg-positive people successfully linked to care who completed a full hepatitis B staging, defined as having all of the following tests: alanine transaminase (ALT), HBV DNA PCR, and FibroScan®; and (iv) the proportion of HBsAg-positive people who completed full clinical staging and who were eligible for anti-HBV therapy. We assessed whether age group, sex, ethnic group, or screening setting is associated with each of these endpoints by using the chi-square test for categorical variables and the Kruskal–Wallis test for continuous variables. Subsequently, a logistic regression was fitted to identify factors associated with each of these endpoints using multivariable analyses mutually adjusted for these variables (age group, sex, ethnic group, and screening setting). To identify factors associated with eligibility for anti-HBV therapy, in addition to the demographic factors described above (age group, sex, ethnic group, and screening setting), we included the following biological variables in the multivariable model: HBeAg positivity, aspartate aminotransferase (AST) levels (<40 or ≥40 IU/ml), gamma-glutamyl transferase (GGT) levels (<60 or ≥60 IU/ml), total bilirubin (<17 or ≥17 IU/ml), and platelet counts (<150 or ≥150 × 10^9^ cells/L). Finally, the 4 continuum-of-care outcomes were presented in percentages using 2 different denominators: 1 constantly using a total number of HBsAg-positive patients as a denominator (*e.g.* proportion of HBsAg-positive patients who were eligible for treatment, irrespective of whether they were linked to care or completed clinical staging) and another using the number of preceding population as a denominator (*e.g.* proportion of those who completed clinical staging who were eligible for treatment).

All the analyses were done using Stata 14.2 (StataCorp, College Station, TX, USA). The study was approved by the National Ethics Committee (SEN11/34).

## Results

### HBV screening

From 1 January 2013 to 31 March 2016, we screened 3,665 individuals in communities (n = 2,153) and workplaces (n = 1,512). Those screened in the communities were mainly females (75.3%, 1,622/2,153) with a median age of 49 years (IQR 39–60), whereas those screened in workplaces were mainly males (78%, 1,179/1,512) with a median age of 43 years (IQR 35–52). The screening uptake was 83.9% (1,512/1,801) in workplaces but could not be evaluated in the community, where the number of people invited to screening was unknown.

**Fig. 1:**
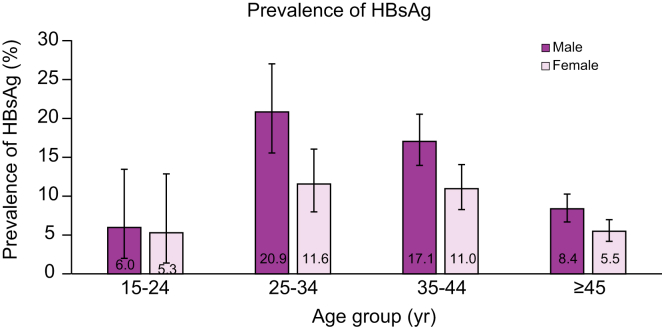
HBV prevalence among people screened in the community and workplaces by sex and age group.

Of the 3,665 individuals screened in the community and workplaces, 366 were tested positive for HBsAg (199 in the community and 167 in workplaces), giving an overall prevalence of 10.0% (95% CI 9.0–11.0). Fig. 1 shows the age- and sex-specific prevalence of both the community and workplace screening. The HBsAg prevalence in the workplaces tended to be higher than that in the community in a crude analysis (11.0% *vs*. 9.2%, *p* = 0.073; Table S1). However, this difference was fully explained by the difference in distribution of age and sex between the community and workplace screening. Table S1 shows that the age-specific prevalence and sex-specific prevalence in each screening setting were quite similar, except for women. Multivariable analysis found that the prevalence of HBsAg significantly varied according to sex and age groups. In contrast, there was no association between the screening setting and HBsAg prevalence after adjusting for age, sex, and ethnic group (Table S2).

During the same period, 638 individuals (median age 33 years, IQR 26–39; male sex 56.4%) who visited the outpatient clinics in the 3 study hospitals were identified to carry HBsAg and enrolled in the study.

### Linkage to HBV care and infection assessment

Table 1 presents the proportion of HBsAg-positive individuals who were successfully linked to care stratified by the screening setting. The proportion successfully linked to care did not differ between the screening sites: 139/199 (69.9%) for the communities, 116/167 (69.5%) for the workplaces, and 463/638 (72.6%) for the hospitals (adjusted *p* = 0.480). Linkage to care was similar irrespective of sex but was higher in the youngest age group of 15–24 years (Table S3).

**Table 1:** **Proportion of HBsAg-positive individuals who were successfully linked to care stratified by the screening setting**.

Variables	All screening settings (N = 1,004)	Community (n = 199)	Workplaces (n = 167)	Hospitals (n = 638)	*p* value∗
Successfully linked to care, n (%)	718/1,004 (71.5)	139/199 (69.9)	116/167 (69.5)	463/638 (72.6)	0.617
Age group, n/N (%) *missing* = *4*					
15–24 years	112/139 (80.6)	0/0 (N/A)	8/9 (88.9)	104/130 (80.0)	0.514
25–34 years	223/315 (70.8)	29/47 (61.7)	19/28 (67.9)	175/240 (72.9)	0.284
35–44 years	230/314 (73.3)	52/66 (78.8)	54/77 (70.1)	124/171 (72.5)	0.481
≥45 years	150/232 (64.7)	58/86 (67.4)	35/53 (66.0)	57/93 (61.3)	0.671
Sex, n/N (%)					
Female	295/423 (69.7)	88/131 (67.2)	11/14 (78.6)	196/278 (70.5)	0.606
Male	423/581 (72.8)	51/68 (75.0)	105/153 (68.6)	267/360 (74.2)	0.396
Ethnicity, n (%) *missing* = *90*					
Wolof	262/373 (70.2)	43/63 (68.3)	55/76 (72.4)	164/234 (70.1)	0.867
Serere	190/269 (70.6)	73/93 (78.5)	19/31 (61.3)	98/145 (67.6)	0.094
Others	185/272 (68.0)	22/41 (53.7)	40/58 (69.0)	123/173 (71.1)	0.097

n.a., not applicable.

∗ *p* value for comparison of prevalence of positive HBsAg between the various screening settings using the chi-squared test.

Among those who were successfully linked to care, 64.6% (464/718) completed the full clinical assessment, defined as completing ALT measurement, HBV viral load, and FibroScan®. This proportion significantly differed between screening settings: full staging was obtained in 47.5% (66/139), 59.5% (69/116), and 71.1% (329/463) of HBV-infected individuals successfully linked to care and originally screened in the community, workplaces, and hospitals, respectively (Table S4). The association between the screening setting and full staging remained significant after adjusting for age, sex, and ethnicity: using community screening as a reference, the odds of completing clinical staging was 1.64 (95% CI 0.93–2.88) in the workplace screening and 3.13 (95% CI 1.98-–4.96) in the hospitals (adjusted *p* <0.001; Table 2). Missing FibroScan® values were the main barrier for complete staging (Table S4).

**Table 2:** **Factors associated with complete hepatitis B staging**.

Variables	Complete HBV staging	cOR (95% CI)	*p* value	aOR (95% CI)	*p* value
Age group, n/N (%)			0.070		0.555
15–24 years	66/103 (64.1)	Ref.		Ref.	
25–34 years	136/202 (67.3)	1.11 (0.67–1.82)		1.01 (0.45–1.62)	
35–44 years	171/250 (68.4)	1.18 (0.72–1.92)		1.32 (0.77–2.24)	
≥45 years	91/162 (56.2)	0.70 (0.42–1.17)		0.94 (0.52–1.70)	
Sex, n/N (%)			0.675		0.915
Female	188/295 (63.7)	Ref.		Ref.	
Male	276/423 (65.3)	1.07 (0.78–1.46)		1.02 (0.71–1.48)	
Ethnicity, n/N (%)			0.198		0.509
Wolof	180/262 (68.7)	Ref.		Ref.	
Serer	115/190 (60.5)	0.70 (0.47–1.03)		0.84 (0.56–1.27)	
Others	120/185 (64.9)	0.84 (0.56–1.25)		0.80 (0.53–1.20)	
Screening setting, n/N (%)			<0.001		<0.001
Community	66/139 (47.5)	Ref.		Ref.	
Workplace	69/116 (59.5)	1.62 (0.99–2.67)		1.64 (0.93–2.88)	
Hospitals	329/463 (71.1)	2.72 (1.84–4.01)		3.13 (1.98–4.96)	

aOR, adjusted odds ratio; cOR, crude odds ratio.

The characteristics of HBV-infected patients who completed the full hepatitis B staging are summarised in Table 3. The vast majority of patients (71.3%, 258/362) were classified as HBeAg-negative chronic HBV infection (previously known as inactive carriers) as defined by normal ALT level (<40 IU/L) and viral load <2,000 IU/ml and none or mild liver fibrosis. The proportion of patients identified as individuals with cirrhosis using LSM was 18.9% (10/53) in workplaces, 6.7% (4/60) in communities, and 11.3% (34/301) in hospitals. At enrolment, no hepatocellular carcinoma (HCC) was diagnosed using liver ultrasound; but 2 patients, screened in the hospital, had decompensated cirrhosis with ascites.

**Table 3:** **Characteristics of HBsAg-positive patients with complete clinical staging**.

Variables	All screening settings (N = 464)	Community (n = 66)	Workplaces (n = 69)	Hospitals (n = 329)	*p* value∗
Age (years), median (IQR)	36 (29–43)	43 (36–51)	42 (37–47)	33 (27–40)	<0.001
Age group, n/N (%)					<0.001
15–24 years	66/464 (14.2)	0/66 (0.0)	3/69 (4.4)	63/329 (19.2)	
25–34 years	136/464 (29.3)	13/66 (19.7)	5/69 (7.3)	118/329 (35.9)	
35–44 years	171/464 (36.9)	26/66 (39.4)	40/69 (58.0)	105/329 (31.9)	
≥45 years	91/464 (19.6)	27/66 (40.9)	21/69 (30.4)	43/329 (13.1)	
Male sex, n (%)	276/464 (59.5)	23/66 (34.9)	63/69 (91.3)	190/329 (57.8)	<0.001
Ethnicity, n (%) *missing* = *49*					<0.001
Wolof	180/464 (43.4)	25/65 (38.5)	30/69 (43.5)	125/281 (44.5)	
Serere	115/464 (27.7)	34/65 (52.3)	12/69 (17.4)	69/281 (24.5)	
Others	120/464 (28.9)	6/65 (9.2)	27/69 (39.1)	87/281 (31.0)	
BMI (kg/m^2^), median (IQR) *missing* = *34*	22.4 (20.2–25.4)	24.2 (20.6–27.1)	23.0 (21.5–25.7)	22.1 (20.0–25.1)	0.035
Ever drank alcohol	36 (7.8%)	5 (7.5)	5 (7.2%)	26 (7.9%)	0.187
HBeAg positive, n/N (%) *missing* = *102*	24/362 (6.6)	1/56 (1.8)	3/59 (5.1)	20/247 (8.1)	0.201
HBV DNA (IU/L), median (IQR)	254 (<5–1421)	252 (<5–1046)	234 (<5–790)	254 (<5–1,731)	0.573
HBV DNA ≥2,000 IU/ml, n/N (%)	100/464 (21.6)	10/66 (15.2)	10/69 (14.5)	80/329 (24.3)	0.077
ALT (IU/ml), median (IQR)	24 (18–32)	22 (17–30)	26 (21–33)	23 (17–32)	0.055
ALT ≥40 IU/ml, n/N (%)	63/464 (13.6)	5/66 (7.6)	12/69 (17.4)	46/329 (14.0)	0.231
AST (IU/ml), median (IQR) *missing* = *31*	28 (22–35)	30 (24–35)	30 (25–36)	27 (22–35)	0.052
GGT (IU/ml), median (IQR) *missing* = *50*	26 (21–37)	24 (19–29)	28 (23–37)	27 (21–38)	0.052
ALP (IU/ml), median (IQR) *missing* = *85*	122 (91–163)	104 (71–129)	117 (84–150)	126 (99–167)	0.004
Total bilirubin (IU/ml), median (IQR) *missing* = *102*	11 (7–15)	12 (9–21)	7 (6–12)	11 (8–15)	0.003
Platelet count (10^9^ cells/L), median (IQR) *missing* = *79*	221 (186–264)	223 (178–277)	213 (175–247)	225 (190–268)	0.299
Liver stiffness† (kPa), median (IQR)	5.8 (4.7–7.5)	5.3 (4.3–6.7)	6.9 (5.4–8.7)	5.8 (4.7–7.3)	0.001
METAVIR score†, n/N (%)					0.236
F0–1 (≤7.8 kPa)	325/414 (79.1)	51/60 (85.0)	36/53 (67.9)	238/301 (79.1)	
F2–3 (7.9–9.4 kPa)	29/414 (9.6)	5/60 (8.3)	7/53 (13.2)	29/301 (9.6)	
F4 (≥9.5 kPa)	34/414 (11.3)	4/60 (6.7)	10/53 (18.9)	34/301 (11.3)	
Unreliable liver stiffness measurement, n/N (%)	50/464 (10.8)	6/66 (9.1)	16/69 (23.2)	28/329 (8.5)	0.001
HBeAg-negative chronic infection phase *missing* = *102*	258/362 (71.3)	46/56 (82.1)	40/59 (67.8)	172/247 (69.6)	0.142
Eligible for treatment, n/N (%)					
EASL (2012)	85/464 (18.3)	6/66 (9.1)	21/69 (30.4)	58/329 (17.6)	0.005
WHO with viral load (2015)	26/464 (5.6)	1/66 (1.5)	4/69 (5.8)	21/329 (6.4)	0.291

ALP, alkaline phosphatase; ALT, alanine transaminase; AST, aspartate aminotransferase; GGT, gamma-glutamyl transferase; WHO, World Health Organization.

∗ *p* value was obtained using the Kruskal–Wallis test for continuous variables and the chi-square test for categorical variables.

† Excluding 50 cases with unreliable measurements.

### HBV treatment eligibility

Among the 464 HBV-infected participants who completed full clinical assessment, 85 (18.3%) were eligible for antiviral therapy according to the EASL criteria and 26 (5.6%) according to the WHO criteria (Table 3). In the crude analysis, treatment eligibility rate significantly differed according to the screening setting being the lowest in participants screened in the community (9.1%, 6/66) and the highest in the workplaces (30.4%, 21/69), whereas it was 17.6% (58/329) in the hospitals (unadjusted *p* = 0.007). In the multivariable analysis (Table S5), the following variables were found to be significantly associated with being eligible for anti-HBV therapy: screening setting other than communities, having positive HBeAg, and lower platelet count (<150 × 10^9^ cells/L).

### Acceptability and adherence to antiviral therapy

All the 85 patients eligible for antiviral therapy initiated TDF 300 mg daily, but 1 patient (1.2%) from the community-based screening declined to be treated. Adherence to treatment was classified as very good in 78/84 (92.8%) but moderate in 6/84 (7.2%) patients. All treated patients had detectable viral load at baseline, and most of them (72/84, 85.7%) had undetectable viral load at Month 12. No serious adverse events were observed, and kidney function, as measured by the estimated glomerular filtration rate (eGFR), was preserved in all patients at 1 year. Treatment interruption at 1 year was observed in 5/84 (6%) patients despite having suspected cirrhosis or advanced liver fibrosis, and ‘not feeling sick’ was the main reason reported for stopping medication.

### HBV continuum of care according to the intervention settings

**Fig. 2:**
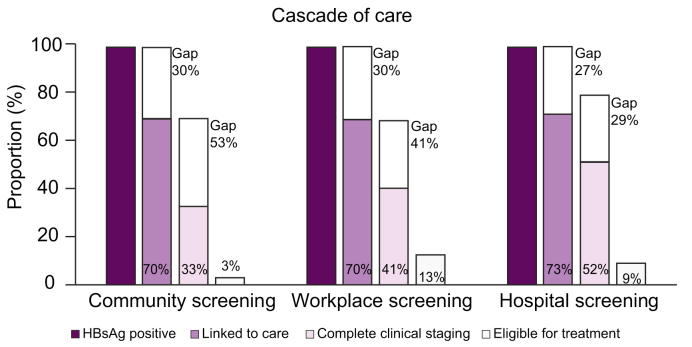
HBV continuum of care by screening setting.

The HBV continuum of care according to the 3 screening settings is summarised in Fig. 2 and Table 4. Although linkage to care of HBV-infected participants was similar according to the intervention setting (69.8% in communities, 69.5% in workplaces, and 72.6% in hospital), the proportion of patients linked to care who completed the full liver assessment differed among the 3 settings (47.5% in communities, 59.5% in workplaces, and 71.1% in hospital). Obtaining full staging among those who linked to care was the most significant gap estimated at 52.5%, 40.5%, and 28.9% in the community-, workplace-, and hospital-based screening interventions, respectively.

**Table 4:** **Continuum of care and gaps observed according to the intervention setting**.

	Community	Workplace	Hospital
Percentage out of HBsAg-positive patients			
HBsAg positive	100% (98.2–100)	100% (97.8–100)	100% (99.4–100)
Linked to care	69.9% (63.0–76.1)	69.5% (61.9–76.3)	72.6% (68.9–76.0)
Complete clinical staging	33.2% (26.7–40.2)	41.3% (33.8–49.2)	51.6% (47.6–55.5)
Eligible for treatment	3.0% (1.1–6.4)	12.6% (7.9–18.6)	9.1% (7.0–11.6)
Percentage out of preceding population			
HBsAg positive	100% (98.2–100)	100% (97.8–100)	100% (99.4–100)
Linked to care	69.9% (63.0–76.1)	69.5% (61.9–76.3)	72.6% (68.9–76.0)
Complete clinical staging	47.5% (39.0–56.1)	59.5% (50.0–68.5)	71.1% (66.7–75.2)
Eligible for treatment	9.1% (3.4–18.4)	30.4% (19.9–42.7)	17.6% (13.7–22.2)
Gaps in care/unmet need∗			
Linked to care	30.2% (23.9–37.0)	30.5% (23.6–38.1)	27.4% (24.0–31.1)
Complete clinical staging	52.5% (43.9–61.0)	40.5% (31.5–50.0)	28.9% (24.8–33.3)

∗ Assuming that the recommended target is 100%.

## Discussion

This real-life intervention study found that HBV screen-and-treat interventions in Senegal are feasible in both non-hospital and hospital settings. However, our study identified major gaps in the HBV continuum of care, especially in obtaining full clinical staging, which is a critical step needed to guide treatment initiation in HBV-infected patients.

Although about two-thirds of HBsAg-positive participants were successfully linked to care, the proportion who had full clinical staging was not satisfactory. A large proportion (25.3%, 182/718) of HBsAg-positive participants with successful linkage to care did not have a FibroScan® performed. During the study period and even at the current time, only 1 non-portable FibroScan® machine, donated by the Prolifica programme, was available throughout the region of Thiès. As a result, patients missed the opportunity to have investigations performed in 1 visit and had to attend an additional visit at another site, which was an important barrier especially for participants who were screened in villages located up to 60 km from the closest hospital. In addition, ALT and HBV viral load measurements were not performed in a substantial number of patients linked to care (17.1% [123/718] and 4.7% [34/718], respectively). Although we could not collect precise data on the reasons for incomplete laboratory analyses, we identified the absence of reagents, loss of samples, and patient refusal to have venipuncture performed as potential barriers. In patients who consented only to a limited blood draw, something that is commonly observed in sSA,^24^ clinicians prioritised viral load measurement over ALT.

Substantial gaps in the HBV continuum of care have been reported in the USA.^25^^,^^26^ However, in sSA, the HBV continuum of care has not been well documented. Compared with other infectious diseases (HIV or HCV), the HBV cascade of care relies on complex algorithms using tests that are not routinely available in sSA.^22^^,^^27^ Our findings suggest an urgent need for simplified clinical algorithms in sSA that would enable a reduction in the number of tests and visits required and therefore minimise loss to follow-up. The development of ALT POC tests^28^ and the use of HBV DNA POC tests^29^^,^^30^ or alternative serological markers^31^^,^^35^ and simple biochemical markers of liver fibrosis might overcome these barriers.^13^ We did not assess liver fibrosis using the AST-to-platelet ratio index (APRI) in this study because it has been shown to have poor diagnostic performance in previous studies in Africa.^20^^,^^32^

In this study, through the community-based screening intervention, we found a high prevalence of HBsAg in the general adult population (9.2%). To the best of our knowledge, this is the first estimate of HBV prevalence in the general adult population in Senegal. The prevalence found here was similar to the prevalence previously reported by our group in the Gambian population with a similar age and sex distribution, with males aged 25–44 years being the most affected by the HBV epidemic.^7^ In The Gambia, the community-based screening resulted in a higher linkage to care (81%), because we provided active outreach clinical staging through a dedicated mobile clinic for those living in remote areas.^7^ In contrast, in this implementation study, the clinical staging was integrated into the locally available healthcare system. People identified to be infected with HBV through the community-based screening were required to travel to the study hospitals although the transportation costs were covered by the study.

Our study fills an important knowledge gap by providing information on the proportion of HBV-infected persons who are eligible for antiviral treatment in sSA. As highlighted by a recent systematic review on HBV treatment eligibility rate globally, data from Africa are scarce and urgently needed.^17^ Importantly, our intervention in workplaces identified a high proportion of participants eligible for antiviral therapy (30.4%) as well as a high proportion of participants with suspected cirrhosis (18.9%). This is certainly related to a high proportion of men aged 25–45 years; male sex was a well-known independent risk factor of liver disease severity^22^ Only a minority reported occasional, nonexcessive alcohol intake. However, in a country where alcohol intake is not well accepted for religious and cultural reasons, care must be taken to interpret the information obtained through a questionnaire.

Treatment eligibility rate observed in the hospital setting (17.6%) was close to the rates reported by other hospital-based studies, for example, in Cameroon (18.4%)^11^ and in Ethiopia (25%).^33^ Importantly, as suggested by other African studies,^7^^,^^8^ antiviral therapy was well accepted with a very good compliance, safety profile, and virological response after 1 year of treatment. At 1 year, we observed 6% of treatment interruption, similar to what was reported in a large cohort of patients with HBV in Ethiopia.^8^

Our study has some limitations. Firstly, it was restricted to the region of Thiès and therefore is not entirely representative of Senegal. Secondly, we were unable to estimate the uptake to screening in the community. However, in the workplaces, the uptake was very good (84%). Thirdly, we did not prospectively collect reasons for incomplete continuum of care. Fourthly, we assessed treatment eligibility at a single time point. Fifthly, we observed overrepresentation of women in people screened for HBV in community settings (1,535 women *vs*. 618 men). Higher screening attendance among women has been also observed in The Gambia^7^ and is likely to be related to a difference in health-seeking behaviour between women and men. Sixthly, we were unable to rule out HCV and HDV co-infections in all patients. However, among those who were tested, only a minority (0.4%) had positive HCV Ab (data not shown). HDV serology is not routinely available in Senegal. Therefore, only a subgroup of patients underwent HDV serology, and about 3% were positive, which is in line with previous estimates in West Africa.^34^ Finally, all clinical and laboratory costs, antiviral therapy, and the transportation fees to attend the clinical appointments were covered by the research programme. As a result, the continuum of care might be an overestimate compared with a real-life setting in sSA where people need to make out-of-pocket payments.

Since January 2016, the Senegalese government has been providing free HBsAg testing and access to low-cost antiviral therapy. However, our study suggests that the implementation of HBV screen-and-treat interventions on a large scale in Senegal will require major simplification of the HBV continuum of care to optimise clinical decision-making and treatment coverage and to eventually achieve HBV elimination.

## Financial support

This work was supported by EC-FP7 (grant 265994) and MRC UK (NIRG).

## Authors’ contributions

Designed the study: MRT, MM, SM, MK, ML, YS. In charge of the study management and the laboratory aspects: AS. Assisted with the study management and the laboratory aspects: GL, OTP, IC, MM, SM, CTK. In charge of the clinical aspects: MK, ST, MD, AK, MO, ML. Contributed to clinical assessment of the patients: GN, SN, JH. Provided support for data collection: LB. In charge of data analysis: YS, JM, ML. Drafted the manuscript: ML, AS, YS. Reviewed and approved the manuscript: All authors.

## Data availability statement

The authors confirm that the data supporting the findings of this study are available, and access to raw data can be made through the corresponding author upon reasonable request.

## Disclaimer

Where authors are identified as personnel of the International Agency for Research on Cancer/WHO, the authors alone are responsible for the views expressed in this article, and they do not necessarily represent the decisions, policy, or views of the International Agency for Research on Cancer/WHO.

## Conflicts of interest

ML, YS, SM, and MT have received consultancy fees and research support from Gilead Sciences.

Please refer to the accompanying ICMJE disclosure forms for further details.
