# Supplementary material for: HBV continuum of care using community- and hospital-based screening interventions in Senegal: Results from the PROLIFICA programme
Source: JHEP Rep. 2022 Jul 9;4(10):100533. doi: 10.1016/j.jhepr.2022.100533 (PMC9424572; doi:10.1016/j.jhepr.2022.100533)
Supplement: Multimedia component 1 [file mmc1.pdf]

HBV continuum of care using community- and hospital-based screening interventions in Senegal

Amina Sow, Maud Lemoine, Papa Souleymane Toure, Madoky Diop[4], Gora Lo[1] Jean De Veiga, Omar Thiaw Pape, Khady Seck, Gibril Ndow, Lamin Bojang, Arame Kane, Marina Oudiane, Jess Howell, Shevanthi Nayagam, Jude Moutchia, Isabelle Chemin, Maimuna Mendy, Coumba Toure-Kane, Mark Thursz, Mourtalla Ka, Yusuke Shimakawa, Souleymane Mboup

Table of contents

Table S1 2

Table S2 3

Table S3 4

Table S4 5

Table S5 6

Variables	Total N = 3,665	Community n = 2,153	Workplaces n = 1,512	*p* value*
**HBsAg positive, n/N (%)**	366/3,665 (10.0)	199/2,153 (9.2)	167/1,512 (11.0)	0.073
**Pos. HBsAg by age group, n/N** (%) *missing* = *3*				
15 – 24 years	9/159 (5.7)	0/0 (N/A)	9/159 (5.7)	-
25 – 34 years	75/477 (15.7)	47/296 (15.9)	28/181 (15.5)	0.905
35 – 44 years	143/1,008 (14.2)	66/530 (12.5)	77/478 (16.1)	0.097
≥ 45 years	139/2,018 (6.9)	86/1,326 (6.5)	53/692 (7.7)	0.323
**Pos. HBsAg by sex, n/N (%)**				
Female	145/1,868 (7.8)	131/1,535 (8.5)	14/333 (4.2)	0.007
Male	221/1,797 (12.3)	68/618 (11.0)	153/1,179 (13.0)	0.226
**Pos. HBsAg by ethnicity, n/N (%)** *missing* = *34*				
Wolof	139/1,359 (10.2)	63/660 (9.6)	76/699 (10.9)	0.420
Serer	124/1,314 (9.4)	93/1,049 (8.9)	31/265 (11.7)	0.159
Others	99/958 (10.3)	41/417 (9.8)	58/541 (10.7)	0.654

Table S1: Prevalence of positive HBsAg stratified by community or workplace setting **p value for comparison of prevalence of positive HBsAg between the various screening settings using chi-squared test*

**Variables**	**Positive HBsAg**	**cOR (95% CI)**	***p* value**	**aOR (95% CI)**	***p* value**
**Age group, n/N (%)**			<0.001		<0.001
15 – 24 years	9/159 (5.7)	Ref.		Ref.	
25 – 34 years	75/477 (15.7)	3.11 (1.52 – 6.37)		3.42 (1.58 – 7.38)	
35 – 44 years	143/1,008 (14.2)	2.76 (1.37 – 5.52)		2.95 (1.40 – 6.23)	
≥ 45 years	139/2,018 (6.9)	1.23 (0.62 – 2.47)		1.32 (0.62 – 2.81)	
**Sex, n/N (%)**			<0.001		<0.001
Female	145/1,868 (7.8)	Ref.		Ref.	
Male	221/1,797 (12.3)	1.67 (1.34 – 2.08)		1.78 (1.37 – 2.31)	
**Ethnicity, n/N (%)**			0.720		0.973
Wolof	139/1,359 (10.2)	Ref.		Ref.	
Serer	124/1,314 (9.4)	0.91 (0.71 – 1.18)		1.01 (0.77 – 1.32)	
Others	99/958 (10.3)	1.01 (0.77 – 1.33)		0.98 (0.74 – 1.29)	
**Screening setting, n/N (%)**			0.074		0.438
Community	199/2,153 (9.2)	Ref.		Ref.	
Workplace	167/1,512 (11.0)	1.22 (0.98 – 1.51)		0.89 (0.68 – 1.18)	

cOR: crude odds ratio, aOR: adjusted odds ratio

Table S2: Factors associated with positive HBsAg

Variables	**Successful linkage to care**	cOR (95% CI)	*p* value	aOR (95% CI)	*p* value
**Age group, n/N (%)**			0.010		0.017
15 – 24 years	112/139 (80.6)	Ref.		Ref.	
25 – 34 years	223/315 (70.8)	0.58 (0.36 – 0.95)		0.57 (0.34 – 0.95)	
35 – 44 years	230/314 (73.3)	0.66 (0.40 – 1.08)		0.74 (0.44 – 1.24)	
≥ 45 years	150/232 (64.7)	0.44 (0.27 – 0.73)		0.47 (0.27 – 0.80)	
**Sex, n/N (%)**			0.288		0.073
Female	295/423 (69.7)	Ref.		Ref.	
Male	423/581 (72.8)	1.16 (0.88 – 1.53)		1.33 (0.97 – 1.82)	
**Ethnicity, n/N (%)**			0.768		0.780
Wolof	262/373 (70.2)	Ref.		Ref.	
Serer	190/269 (70.6)	1.02 (0.72 – 1.43)		1.01 (0.71 – 1.82)	
Others	185/272 (68.0)	0.90 (0.64 – 1.26)		0.89 (0.64 – 1.26)	
**Screening setting, n/N (%)**			0.617		0.480
Community	139/199 (69.9)	Ref.		Ref.	
Workplace	116/167 (69.5)	0.98 (0.63 – 1.54)		0.76 (0.45 – 1.25)	
Hospitals	463/638 (72.6)	1.14 (0.81 – 1.62)		0.81 (0.54 – 1.19)	

cOR: crude odds ratio, aOR: adjusted odds ratio

Table S3. Factors associated with successful linkage to care

Variables	All screening settings n = 718	Community<br>n= 139	Workplace<br>n= 116	Hospitals<br>n = 463	*p* value*
**†Complete clinical staging, n/N (%)**	464/718 (64.6)	66/139 (47.5)	69/116 (59.5)	329/463 (71.1)	<0.001
Age group, n/N (%) *missing = 1*					
15 – 24 years	66/102 (64.7)	0/0 (N/A)	3/7 (42.9)	63/95 (66.3)	0.210
25 – 34 years	136/203 (67.0)	13/26 (50.0)	5/13 (38.5)	118/164 (72.0)	0.007
35 – 44 years	171/250 (68.4)	26/53 (49.1)	40/57 (70.2)	105/140 (75.0)	0.002
≥ 45 years	91/162 (56.2)	27/60 (45.0)	21/39 (53.9)	43/63 (68.3)	0.032
**Sex, n/N (%)**					
Female	188/295 (63.7)	43/88 (48.9)	6/11 (54.6)	139/196 (70.9)	0.001
Male	276/423 (65.3)	23/51 (45.1)	63/105 (60.0)	190/267 (71.2)	0.001
**§Partial clinical staging, n/N (%)**	254/718 (35.4)	73/139 (52.5)	47/116 (40.5)	134/463 (28.9)	<0.001
VL & ALT only	111/254 (43.7)	29/73 (39.7)	17/47 (36.2)	65/134 (48.5)	
VL & FS only	42/254 (16.5)	19/73 (26.0)	5/47 (10.7)	18/134 (13.4)	
ALT & FS only	16/254 (6.3)	0/73 (0.0)	1/47 (2.1)	15/134 (11.2)	
VL only	67/254 (26.4)	20/73 (27.4)	23/47 (48.9)	24/134 (17.9)	
ALT only	4/254 (1.6)	0/73 (0.0)	0/47 (0.0)	4/134 (3.0)	
FS only	14/254 (5.5)	5/73 (6.9)	1/47 (2.1)	8/134 (6.0)	

**p* value for comparison of proportion of clinical staging between the various screening settings using chi-squared test

†VL & FS & ALT done

§At least one of VL, FS, ALT not done

Table S4. Proportion of HBsAg-positive individuals successfully linked to care who had complete clinical staging stratified by the screening setting

Variables	Eligible for treatment	cOR (95% CI)	*p* value	aOR (95% CI)	*p* value
**Age group, n/N (%)**			0.618		0.914
15 – 24 years	14/66 (21.2)	Ref.		Ref.	
25 – 34 years	23/136 (16.9)	0.76 (0.36 – 1.59)		0.69 (0.24 – 1.96)	
35 – 44 years	28/171 (16.4)	0.73 (0.36 – 1.49)		0.74 (0.21 – 2.53)	
≥ 45 years	20/91 (22.0)	1.05 (0.48 – 2.26)		0.72 (0.16 – 3.19)	
**Sex, n/N (%)**			<0.001		0.109
Female	19/188 (10.1)	Ref.		Ref.	
Male	66/276 (23.9)	2.80 (1.61 – 4.84)		2.25 (0.83 – 6.10)	
**Ethnicity, n/N (%)**			0.292		0.596
Wolof	32/180 (17.8)	Ref.		Ref.	
Serer	18/115 (15.7)	0.86 (0.46 – 1.61)		1.10 (0.37 – 3.25)	
Others	28/120 (23.3)	1.41 (0.80 – 2.49)		1.58 (0.63 – 3.93)	
**Screening setting, n/N (%)**			0.007		0.032
Community	6/66 (9.1)	Ref.		Ref.	
Workplace	21/69 (30.4)	4.38 (1.64 – 11.70)		10.13 (1.08 – 94.66)	
Hospitals	58/329 (17.6)	2.14 (0.88 – 5.19)		3.24 (0.34 – 30.61)	
**HBeAg, n/N (%)**			<0.001		0.005
Negative	51/338 (15.1)	Ref.		Ref.	
Positive	16/24 (66.7)	11.25 (4.57-27.66)		11.30 (2.11-60.29)	
**AST, n/N (%)**			<0.001		0.066
< 40 IU/mL	49/365 (13.4)	Ref.		Ref.	

≥ 40 IU/mL	32/68 (47.1)	5.73 (3.26-10.06)		2.44 (0.94-6.32)	
**GGT, n/N (%)**			<0.001		0.180
< 60 IU/mL	59/377 (15.7)	Ref.		Ref.	
≥ 60 IU/mL	17/37 (46.0)	4.58 (2.26-9.26)		2.20 (0.69-6.96)	
**Total bilirubin, n/N (%)**			0.880		0.897
< 17 IU/mL	55/282 (19.5)	Ref.		Ref.	
≥ 17 IU/mL	15/80 (18.8)	0.95 (0.50-1.79)		1.06 (0.41-2.72)	
**Platelet counts, n/N (%)**			<0.001		<0.001
≥ 150 x 10^9 cells/L	46/343 (13.4)	Ref.		Ref.	
< 150 x 10^9 cells/L	20/42 (47.6)	5.86 (2.97-11.59)		8.60 (2.97-24.85)	

Table S5. Factors associated with treatment eligibility using the 2012 EASL criteria
